# Integrating Peripheral Nerve Blocks in Multiple Trauma Care: Current Evidence and Clinical Challenges

**DOI:** 10.3390/jcm14155598

**Published:** 2025-08-07

**Authors:** Liliana Mirea, Ana-Maria Dumitriu, Cristian Cobilinschi, Răzvan Ene, Raluca Ungureanu

**Affiliations:** 1 Faculty of Medicine, “Carol-Davila” University of Medicine and Pharmacy, 050474 Bucharest, Romania; llmirea@yahoo.com (L.M.); cristian.cobilinschi@umfcd.ro (C.C.); razvan.ene@umfcd.ro (R.E.); ralucaung@yahoo.com (R.U.); 2 Anesthesiology and Intensive Care Clinic, Clinical Emergency Hospital, 050098 Bucharest, Romania; 3 Orthopedics and Trauma Surgery, Clinical Emergency Hospital, 050098 Bucharest, Romania

**Keywords:** peripheral nerve blocks (PNBs), polytrauma, pain management, multimodal analgesia, opioid-sparing strategies, enhanced recovery, compartment syndrome

## Abstract

Pain management in multiple trauma patients presents a complex clinical challenge due to competing priorities such as hemodynamic instability, polypharmacy, coagulopathy, and the urgency of life-saving interventions. In this context, peripheral nerve blocks (PNBs) are increasingly recognized as a valuable asset for their role in managing pain in patients with multiple traumatic injuries. By reducing reliance on systemic opioids, PNBs support effective pain control and facilitate early mobilization, aligning with enhanced recovery principles. This narrative review summarizes current evidence on the use of PNBs in the context of polytrauma, focusing on their analgesic efficacy, integration within multimodal analgesia protocols, and contribution to improved functional outcomes. Despite these advantages, clinical application is limited by specific concerns, including the potential to mask compartment syndrome, the risk of nerve injury or local anesthetic systemic toxicity (LAST), and logistical barriers in acute trauma settings. Emerging directions in the field include the refinement of ultrasound-guided PNB techniques, the expanded use of continuous catheter systems, and the incorporation of fascial plane blocks for anatomically complex or multisite trauma. Parallel efforts are focusing on the development of decision-making algorithms, improved risk stratification tools, and integration into multimodal analgesic pathways. There is also growing emphasis on standardized clinical protocols, simulation-based training, and patient education to enhance safety and consistency in practice. As evidence continues to evolve, the long-term impact of PNBs on functional recovery, quality of life, and healthcare utilization must be further explored. With thoughtful implementation, structured training, and institutional support, PNBs may evolve into a cornerstone of modern trauma analgesia.

## 1. Introduction

Effective pain management in patients with multiple trauma is essential not only for patient comfort but also for reducing physiological stress, supporting early mobilization, and minimizing complications such as respiratory compromise, delirium, and thromboembolic events [1,2]. In polytrauma patients, analgesia can be particularly complex due to the interplay of hemodynamic instability, altered mental status, coagulopathy, and the competing demands of acute diagnostic and resuscitative care.

Systemic analgesics, particularly opioids, are fundamental for managing trauma-related pain, but their use is frequently limited by adverse effects. While opioids, NSAIDs, and acetaminophen form the core of pain management, opioids can cause respiratory depression, ileus, delirium, and dependence, especially in vulnerable populations [3]. Furthermore, systemic agents may not adequately address focal pain sources like long bone fractures, rib injuries, or pelvic trauma [4,5,6].

In this context, regional anesthesia is increasingly recognized as a valuable adjunct or alternative to systemic analgesia. PNBs provide targeted, site-specific pain relief, reduce opioid consumption, and may facilitate earlier mobilization and improved functional recovery [7,8]. Their role in trauma care is supported by advances in ultrasound technology, which have increased block accuracy and safety while making them feasible in prehospital, emergency department, and perioperative settings [9,10].

Specific regional techniques such as femoral nerve, fascia iliaca, serratus anterior plane, and brachial plexus blocks have shown efficacy in reducing pain scores and improving patient satisfaction across various trauma subpopulations [9,11]. PNBs have also been integrated into multimodal analgesia strategies and enhanced recovery after surgery (ERAS) protocols in both elective and emergency orthopedic trauma [12].

Despite their clear benefits, the adoption of PNBs in trauma remains inconsistent. Concerns persist regarding the potential masking of evolving neurologic conditions such as compartment syndrome, especially in lower limb trauma [13]. Additional challenges include coagulopathy, limited access to ultrasound equipment, lack of training, and institutional variability in protocols [14,15].

This review aims to summarize the current evidence supporting the use of peripheral nerve blocks (PNBs) in patients with multiple trauma, highlight key clinical challenges, including safety considerations and contraindications, and explore emerging techniques, educational strategies, and future directions for the broader and safer implementation of PNBs in trauma care.

## 2. Current Evidence for Peripheral Nerve Blocks in Trauma Care

As aforementioned, PNBs represent a valuable modality within modern trauma analgesia, offering site-specific pain control while reducing reliance on systemic medications. Their role is well-established in orthopedic and perioperative practice, and emerging evidence supports their use in trauma care to improve pain control while minimizing systemic drug burden [16,17].

### 2.1. Clinical Evidence from Randomized Trials and Meta-Analyses

Numerous randomized controlled trials (RCTs) and meta-analyses support the use of PNBs in traumatic injuries. Femoral nerve blocks (FNBs) and fascia iliaca compartment blocks (FICBs) are among the most extensively studied regional techniques in trauma care, particularly for femoral fractures [18]. These blocks have consistently demonstrated significant reductions in opioid consumption, improved pain scores, and fewer opioid-related adverse effects when compared to systemic analgesia alone [19,20]. In patients with rib fractures, paravertebral blocks are associated with improved respiratory parameters and a lower incidence of pulmonary complications [21,22].

Upper extremity trauma also benefits from regional techniques, including interscalene, supraclavicular, and infraclavicular blocks, which have been shown to provide dense analgesia with minimal systemic effects. These benefits are most pronounced when PNBs are initiated early in the care pathway, such as in the emergency department or prehospital environment [23,24]. In the ED setting, ultrasound-guided regional anesthesia (UGRA) has been increasingly used to facilitate painful procedures—such as closed reductions of dislocations and fractures—without the need for systemic sedation. When performed by trained emergency physicians, blocks such as suprascapular, interscalene, and supraclavicular for upper limb injuries, or popliteal sciatic and posterior tibial nerve blocks for lower limb procedures, offer effective and well-tolerated alternatives. The use of short-acting local anesthetics (e.g., lidocaine 1.0–2%, mepivacaine 1–1.5% and chloroprocaine 2–3%) ensures timely onset and recovery, supporting safe discharge planning while minimizing the risk of masking potential compartment syndrome in patients requiring surgery. UGRA has also proven valuable for hand injuries, where distal forearm blocks targeting the median, ulnar, or radial nerves provide rapid, localized pain control with high patient satisfaction and minimal complications [25,26].

Commonly used peripheral nerve blocks (PNBs) in trauma care are summarized in the table below, highlighting their primary indications, key advantages, and relevant limitations (Table 1).

### 2.2. Guidelines and Society Recommendations

The growing endorsement of peripheral nerve blocks (PNBs) in clinical guidelines marks a significant milestone in trauma pain management, aligning with the broader transformation of trauma care. The last decade has seen a shift toward more individualized, physiology-driven strategies, including improved transfusion practices, metabolic interventions, and targeted hemodynamic management in polytrauma settings [29,30,31].

Although the European Society of Regional Anaesthesia (ESRA) and the American Society of Regional Anesthesia and Pain Medicine (ASRA) primarily address regional techniques in the context of acute and perioperative pain, their published principles—particularly regarding safety, patient selection, and ultrasound guidance—are increasingly applied to trauma populations [32,33]. Trauma-specific systems, such as the London Major Trauma Network, actively integrate PNBs into standardized protocols for select injuries [34]. The French Society of Anesthesiology (SFAR) published an important national consensus on geriatric trauma care, specifically recommending the early implementation of peripheral nerve blocks (PNBs) for femoral fractures, with appropriate monitoring and consideration of contraindications [35]. Together, these developments reflect the growing recognition of PNBs not merely as adjuncts but as critical tools in modern trauma analgesia and systemic care.

## 3. Special Considerations in Multiple Trauma

### 3.1. Hemodynamic Instability and Block Choices

Hemodynamic instability is common among trauma patients due to hypovolemia or hemorrhagic shock. Although PNBs generally have less impact on sympathetic tone compared to neuraxial anesthesia, certain deep blocks such as lumbar plexus or high thoracic paravertebral blocks may still provoke hypotension [28]. Therefore, distal limb blocks, including femoral, sciatic, and brachial plexus blocks, are preferred in unstable patients as they tend to have minimal autonomic involvement [36]. Additionally, altered pharmacokinetics in patients with poor perfusion or organ dysfunction necessitate cautious dosing and careful monitoring for signs of systemic local anesthetic toxicity [37]. Continuous cardiovascular monitoring during block placement is essential to promptly detect and manage any hemodynamic changes.

### 3.2. Neurologic Assessment Limitations Post-Block

A major concern in trauma patients receiving regional anesthesia is the potential masking of evolving neurologic deficits. Spinal cord injuries, nerve ischemia, or compression injuries may go unnoticed if sensory or motor examinations are obscured by the block [38]. Additionally, although rare, peripheral nerve blocks themselves carry a risk of block-related neurologic complications—including mechanical nerve injury, intraneural injection, or ischemic insult—particularly in anatomically distorted or unstable patients [39]. Therefore, a thorough baseline neurologic examination before block placement is critical to establish a reference for subsequent assessments.

In patients with suspected nerve trauma or “double crush” phenomena—a condition discussed in more detail in the following section—PNBs may complicate clinical differentiation of injury progression [40]. To mitigate this risk, modified approaches such as single-shot blocks with short-acting local anesthetics or low-concentration continuous infusions can preserve partial sensory function, allowing ongoing neurologic monitoring [41]. Frequent and detailed post-block neurologic assessments are mandatory to detect any deterioration promptly.

### 3.3. Anticoagulation and Bleeding Risk

Anticoagulation is common in trauma patients for thromboprophylaxis, treatment of embolism, or management of vascular injuries, and it significantly influences the safety of PNBs [42]. Superficial blocks, such as femoral or interscalene blocks, are generally considered safe even in anticoagulated patients due to the low risk of bleeding complications. Conversely, deeper blocks like lumbar plexus, parasacral sciatic, or infragluteal approaches carry a higher risk of serious bleeding, including retroperitoneal hematoma or nerve compression, and therefore should be avoided or timed carefully in relation to anticoagulant administration [43]. Adherence to established guidelines from professional societies such as ASRA, ESRA and the European Society of Anaesthesiology and Intensive Care (ESAIC) is essential for timing block placement relative to specific anticoagulants. Additionally, patients require close post-procedure monitoring for signs of bleeding or nerve dysfunction.

### 3.4. Polytrauma Logistics: Timing, Positioning, and Imaging

Logistical challenges in polytrauma patients further complicate the timing and feasibility of PNB placement. The need to prioritize life-saving interventions often limits the window available for regional anesthesia. While early block placement can improve pain control and facilitate patient cooperation for diagnostic imaging, it should never delay critical trauma assessments or resuscitation [44]. Positioning restrictions due to spinal precautions, fractures, or immobilization devices may impede access to certain nerves, such as the sciatic nerve, which often requires lateral decubitus positioning. In these cases, the use of ultrasound guidance is invaluable to improve block accuracy and safety, particularly when anatomical landmarks are distorted by swelling, hematomas, or dressings [45]. Successful analgesic management requires close interdisciplinary collaboration among trauma surgeons, anesthesiologists, orthopedic surgeons, and emergency care providers to integrate PNBs effectively within the broader trauma treatment plan.

## 4. Challenges in Clinical Practice

### 4.1. Delayed Diagnosis of Compartment Syndrome

The use of PNBs in trauma patients raises concerns regarding the potential delay in diagnosing compartment syndrome. PNBs, especially continuous infusions or dense motor and sensory blocks, may obscure classical symptoms such as disproportionate pain, paresthesias, and motor deficits. Proponents of PNBs note that with proper clinical vigilance and the use of low-concentration local anesthetics (e.g., ropivacaine 0.1–0.2%), early signs like increased pain on passive stretch may still be evident. Additionally, evidence suggests that ischemic pain is not completely abolished by PNBs; compartment syndrome often manifests through breakthrough pain, escalating opioid needs, and physical exam findings despite the block [46,47]. In fact, multiple case reports have documented that the presence of severe breakthrough pain despite functioning nerve blocks served as an early indicator of evolving compartment syndrome, prompting timely intervention.

Conversely, retrospective case reports have documented delayed diagnoses in patients receiving regional anesthesia, particularly in situations where clinical monitoring was inadequate [48]. The ASRA–ESRA joint practice advisory addresses this complexity by stating that PNBs are not contraindicated in suspected compartment syndrome, provided that clinicians implement rigorous monitoring protocols and remain alert to the possibility of symptom masking [49]. Best-practice recommendations emphasize avoiding continuous local anesthetic infusions in compartments at high risk, preferring short-acting agents, and maintaining vigilant clinical surveillance. It is also recommended to provide clear post-discharge instructions—such as written guidance, follow-up phone calls, and access to medical contact—in ambulatory cases involving patients at risk. Importantly, the current literature remains limited to case series and case reports (Class IV evidence), and no randomized controlled trials exist to definitively assess the risk of PNB-related delays in ACS diagnosis. Despite this, when combined with proper clinical protocols, regional anesthesia remains a valuable and safe component of trauma pain management [50].

### 4.2. Double Crush Syndrome

Double crush syndrome describes a condition where a nerve already compromised at one site—for example, by cervical radiculopathy—is more vulnerable to additional injury at a second site, such as limb trauma [40]. This phenomenon presents a particular challenge in multiple trauma patients, where nerve stretch, contusion, or traction injuries are common.

Administering a PNB to a nerve with a pre-existing injury introduces two significant concerns [51]. Firstly, the block may mask neurological deficits present before the trauma, complicating baseline assessment and post-operative evaluation. Secondly, some evidence suggests that anesthetizing an already compromised nerve could impair recovery or prolong dysfunction, although direct causality remains uncertain. While double crush syndrome is not an absolute contraindication to PNBs, many experts advocate caution. Strategies such as using lower concentrations of local anesthetic, shorter-acting agents, or even avoiding PNBs altogether in cases of documented nerve injury are prudent [52]. This approach aims to minimize the potential exacerbation of nerve dysfunction while still providing effective analgesia.

### 4.3. High-Flow Trauma Settings

The implementation of regional anesthesia in high-flow or acute trauma settings presents notable logistical challenges. Time pressures, limited access to ultrasound equipment, and shortages of trained personnel frequently result in a preference for systemic analgesia during the initial stabilization phase [26]. The fast-paced and often chaotic environments of emergency departments and trauma bays may not be conducive to the detailed attention and time required for safe, effective peripheral nerve block placement [53].

Despite these obstacles, delayed or secondary application of PNBs—after the primary survey and initial resuscitation—has gained acceptance as a valuable strategy for pain control with significant opioid-sparing benefits. The presence of an acute pain service (APS) or a dedicated regional anesthesia team available on-call can improve the timely access to PNBs, even in busy trauma settings, thereby optimizing analgesia without compromising patient safety [54].

Furthermore, the increased availability and adoption of ultrasound guidance (US) in these settings has enhanced the success rates and safety profile of PNBs. Our review and other large-scale data [55] suggest that ultrasound guidance reduces the incidence of nerve injury, particularly in blocks with higher baseline risk such as interscalene approaches, by allowing real-time visualization of nerve structures, needle placement, and local anesthetic spread. However, the benefits of US guidance are operator-dependent and require adequate training and equipment availability, which remain challenges in high-flow trauma settings.

### 4.4. Training and Operator Dependency

The safety and effectiveness of peripheral nerve blocks depend heavily on operator skill and the availability of institutional resources. Performing high-quality PNBs requires detailed anatomical knowledge, ultrasound proficiency, and the ability to interpret patient responses during block placement [56]. These skills may not be uniformly available across trauma centers, especially those lacking robust anesthesiology support.

Many trauma centers face gaps in standardized training protocols and simulation-based teaching programs for regional anesthesia [57]. Consequently, complication rates may be higher, and block failure or the underutilization of PNBs may occur when less-experienced personnel perform procedures without adequate supervision. Although neurological complications after PNBs are uncommon and their occurrence has declined over time—probably owing to the wider use of ultrasound guidance and advancements in technique—these improvements rely heavily on ongoing training and strict adherence to protocols. The differences in complication rates among various nerve blocks may also be influenced by anatomical variations and the skill level of the operator, highlighting the importance of customized education and training programs [55].

Addressing these challenges involves developing trauma-specific PNB algorithms, implementing simulation workshops, and standardizing protocols. The use of pre-packed block kits and checklists can streamline urgent block placement. Furthermore, fostering interdepartmental collaboration among emergency, orthopedic, and anesthesia teams may enhance both the adoption and safety of regional anesthesia techniques in trauma care.

## 5. Integration with Multimodal Analgesia

### 5.1. PNBs Within Multimodal Trauma Protocols and ERAS

Multimodal analgesia is a cornerstone of contemporary trauma management aimed at minimizing opioid reliance while optimizing pain control and promoting functional recovery. Although ERAS principles were initially developed for elective surgery, they are increasingly being tailored to trauma pathways, particularly in orthopedic trauma and rib fracture care [58,59]. Peripheral nerve blocks serve as foundational modalities in these protocols; blocks such as the femoral nerve block, fascia iliaca compartment block, and paravertebral blocks have demonstrated significant efficacy in managing pain from hip fractures, rib fractures, and upper limb injuries. Their use has been associated with improved early mobilization and reduced opioid consumption. As a result, many trauma centers have incorporated early PNBs into standardized bundled care pathways, especially targeting elderly patients with fragility fractures or those suffering polytrauma involving the extremities [60,61].

### 5.2. Combination with Systemic Non-Opioid Analgesics

PNBs are often combined safely and effectively with systemic non-opioid analgesics to optimize pain control. Low-dose ketamine infusions are frequently used adjuncts in trauma patients, particularly those with opioid tolerance, hypotension, or severe pain. When used alongside PNBs, ketamine enhances analgesia by reducing central sensitization and improving pain control [62]. Additionally, acetaminophen and non-steroidal anti-inflammatory drugs (NSAIDs) provide baseline anti-inflammatory and analgesic effects, complementing the peripheral nerve block to reduce breakthrough pain and potentially facilitate earlier functional recovery in the acute post-injury period [63]. This multimodal approach enables the individualized titration of pain management based on patient-specific factors and injury severity, while minimizing the risk of sedation or respiratory depression.

## 6. Emerging Techniques and Technologies

The evolution of peripheral nerve block techniques in trauma care has been propelled by advances in imaging, equipment, and anatomical understanding. These innovations aim to improve block accuracy, expand indications in complex trauma cases, and enhance safety through continuous monitoring and precision delivery.

### 6.1. Ultrasound-Guided PNB in Trauma

Ultrasound (US) guidance has revolutionized regional anesthesia, especially in trauma settings. It provides the real-time visualization of nerves, adjacent structures, and local anesthetic spread—an essential advantage when anatomical landmarks are distorted by injury, swelling, or immobilization. In trauma care, US guidance increases first-attempt success rates, reduces risks such as vascular puncture or nerve injury, and enables safer performance in coagulopathic patients by avoiding deeper vascular planes [64]. This technique is particularly valuable in patients with multiple injuries, where traditional landmark-based approaches may be unsafe or impractical [65]. Recent evidence from a systematic review and meta-analysis has further informed practice regarding the use of local anesthetic (LA) mixtures in ultrasound (US)-guided peripheral nerve blocks. The findings indicate that mixing LAs offers no clear benefit in accelerating sensory or motor onset. Instead, it may shorten sensory block duration without improving 24 h pain scores or reducing opioid use. Given the uncertain safety profile and inconsistent findings, LA mixtures should be used cautiously in trauma settings, where single-agent, long-acting LAs may provide more predictable and sustained analgesia [66].

### 6.2. Continuous Peripheral Nerve Block Techniques

Continuous catheter-based PNB techniques are increasingly utilized for prolonged analgesia in patients with major limb trauma, multiple rib fractures, or staged surgeries. Common targets include femoral, sciatic, infraclavicular, and interscalene nerves, with expanding use of paravertebral and erector spinae plane (ESP) catheters for thoracic injuries [64,67]. These catheters allow precise titration or the discontinuation of local anesthetics in response to neurologic changes, enhancing safety. However, securing catheter placement and preventing infection are critical, particularly in austere or field environments where monitoring may be limited [68].

Continuous peripheral nerve blocks offer the advantage of sustained analgesia, which can be titrated based on patient needs by adjusting infusion rates or local anesthetic concentration. This flexibility allows for prolonged pain control, opioid-sparing effects, and improved functional recovery, particularly in complex trauma cases requiring extended analgesia. Typical dosing regimens involve low-concentration local anesthetics such as ropivacaine 0.1–0.2% or bupivacaine 0.1–0.125%, infused at rates ranging from 5 to 10 mL/h, sometimes combined with intermittent boluses to optimize spread and efficacy [69].

Nevertheless, continuous techniques are not without risks—catheter dislodgement, infection, and local anesthetic systemic toxicity can occur, especially if dosing is not carefully managed. In contrast, single-shot (bolus) PNBs provide effective short-term analgesia with fewer equipment-related complications and simpler implementation, making them attractive in high-flow or resource-limited settings. However, their limited duration may necessitate repeat interventions or adjunctive systemic analgesia [70].

Ultimately, the choice between continuous and bolus PNBs should be individualized based on patient factors, injury complexity, the expected duration of pain, and institutional resources.

### 6.3. Fascial Plane Blocks in Complex Trauma

Newer fascial plane blocks provide practical alternatives when traditional peripheral nerve blocks (PNBs) are contraindicated, technically challenging, or anatomically unfeasible. The erector spinae plane (ESP) block has demonstrated effectiveness in providing analgesia and reducing opioid requirements in patients with rib fractures as well as thoracic, lumbar, or abdominal trauma [71]. The serratus anterior plane block (SAPB) is particularly useful for lateral thoracic trauma, such as rib fractures [72,73]. PECS I and II blocks may be valuable in select cases of anterolateral chest wall injuries involving soft tissue, especially following blunt trauma or surgical incisions [74].

The quadratus lumborum (QL) block provides somatic—and possibly visceral—analgesia for lower abdominal and pelvic injuries [75]. The transversus abdominis plane (TAP) block is another option for abdominal wall analgesia [76], serving as a valuable component of multimodal strategies aimed at reducing postoperative surgical stress response, complementing well-established approaches in major thoraco-abdominal procedures [77]. Additionally, the pericapsular nerve group (PENG) block is an emerging targeted interfascial plane block designed to provide effective analgesia for hip fractures and anterior hip pain by anesthetizing the articular branches of the femoral nerve, accessory obturator nerve, and obturator nerve [78]. Like other fascial plane blocks, it is performed under ultrasound guidance and offers the advantages of being motor-sparing and relatively superficial, making it particularly suited for trauma patients where preserving mobility is important [79,80].

These fascial plane blocks tend to be superficial, are easily performed under ultrasound guidance, carry a lower risk of motor blockade or hemodynamic instability, and may be safer in anticoagulated patients depending on block depth and site.

### 6.4. Smart Pumps, Infusion Monitoring, and Nerve Block Mapping

Technological advancements are increasingly integrated into regional anesthesia practice to enhance safety and individualization. Emerging tools include smart infusion pumps, which enable the precise titration of local anesthetic dosing and monitoring of infusion patterns, as well as digital platforms that enable the real-time visualization and documentation of nerve blocks [81]. Nerve block mapping platforms are emerging, allowing automated nerve recognition and block planning, potentially expanding accessibility and improving patient safety [82]. In addition to nerve identification, ultrasound automation may offer real-time needle trajectory guidance and smart safety alerts for proximity to vessels or pleura. These technologies could lower technical barriers and expand PNB availability, especially in resource-limited or prehospital settings.

## 7. Future Directions

Despite the growing integration of PNBs into trauma care, critical gaps remain in the evidence base and clinical standardization. Addressing these gaps through rigorous research, protocol development, and technology integration is essential to optimize patient outcomes and expand safe, effective analgesic options for this complex patient population.

Most existing data on PNBs come from elective surgeries or isolated injuries, with limited research specifically addressing polytrauma populations. High-quality RCTs are needed to evaluate pain control, functional recovery, complication rates, ICU length of stay, and long-term neurological outcomes in this complex cohort.

Trauma teams would benefit from structured decision-making algorithms incorporating injury pattern, hemodynamic stability, coagulopathy, patient consciousness, and institutional expertise. Embedding such protocols into trauma care pathways could improve block selection and patient safety.

As the use of peripheral nerve blocks (PNBs) expands, standardized skills for anesthesiologists, emergency physicians, and acute care surgeons becomes essential. Simulation-based training tailored to trauma-specific challenges—like unstable patients, altered anatomy, and austere environments—is crucial for skill development. Implementing institutional protocols that include pre-prepared kits and checklists can help minimize variability and procedural delays.

## 8. Conclusions

Peripheral nerve blocks represent a powerful analgesic tool of multimodal pain management in trauma patients, enabling targeted analgesia that complements systemic therapies. Their integration into comprehensive care pathways supports improved patient comfort and functional recovery. Despite their proven benefits, challenges remain in safely integrating PNBs into complex polytrauma management, especially given concerns around neurologic monitoring, coagulopathy, and logistical constraints.

Advancements in ultrasound guidance, continuous catheter techniques, and emerging technologies are expanding the feasibility and safety of PNBs in diverse trauma settings. However, broader adoption depends on standardized clinical protocols, multidisciplinary collaboration, and robust training programs tailored to the unique demands of trauma patients.

Future research focusing specifically on polytrauma populations, incorporating long-term outcomes and patient-centered metrics, is essential to refine best practices. By addressing current knowledge gaps and embracing technological innovations, trauma care teams can optimize analgesic strategies—improving both immediate pain control and long-term recovery for this vulnerable patient group.

## Figures and Tables

**Table 1:** Regional techniques for acute trauma analgesia [18,24,25,26,27,28].

Block	Indications	Advantages	Limitations/Concerns
**Fascia Iliaca Block** **(FICB)**	Hip, femur, and acetabular fractures	Easy, effective, and safe in ED	Usually miss obturator nerve
**Femoral Nerve Block** **(FNB)**	Mid-shaft femur and patella	Rapid relief and opioid-sparing	Motor block may delay rehab
**Sciatic Nerve Block**	Tibial/fibular fractures	Excellent distal coverage Popliteal approach preserves knee flexion	Requires expertise
**Paravertebral**	Multiple rib fractures	Good thoracic analgesia	Technically demanding
**Interscalene/Supraclavicular Block**	Upper limb trauma	Fast onset and good density	Risk of phrenic block
